# Illumination on Chemical Compounds from Qufeng Zhitong Capsule and Its Potential Pharmacological Mechanism against Rheumatoid Arthritis Based on UHPLC/Q-Orbitrap-MS Combined with Network Pharmacology Analysis

**DOI:** 10.1155/2022/7863435

**Published:** 2022-12-07

**Authors:** Mengjie Xue, Lihua Zhang, Yuting Zhao, Qixuan Mu, Ying Cui, Ke Qian, Xin Chai

**Affiliations:** ^1^State Key Laboratory of Component-based Chinese Medicine, Tianjin Key Laboratory of TCM Chemistry and Analysis, Tianjin University of Traditional Chinese Medicine, Tianjin 301617, China; ^2^Shaanxi Buchang Pharmaceutical Co., Ltd., Xianyang, Shaanxi 712000, China; ^3^Haihe Laboratory of Modern Chinese Medicine, Tianjin 301617, China

## Abstract

Qufeng Zhitong capsule (QZC), a Chinese patent medicine officially approved in China for the treatment of rheumatoid arthritis (RA) and other diseases, possesses the primary effects of dispelling wind, relieving pain, and promoting blood circulation, whose clinical applications have been confined owing to the incomplete elucidation of its chemical compositions and the underlying molecular mechanism for the treatment of RA. In this study, 61 compounds including 16 phenylpropanoids, 15 organic acids, 13 alkaloids, seven flavonoids, six iridoids, one saccharide, two aldehydes, and one saponin in QZC were simultaneously identified and traced to their herbal origins by ultra-high performance liquid chromatography tandem Q-Exactive Orbitrap high-resolution mass spectrometry (UHPLC/Q-Orbitrap-MS), where 31 of them were unambiguously identified by reference compounds, and the other 30 were tentatively characterized. Besides, all these compounds were proven to have potential pharmacological activity in the treatment of RA based on network pharmacology analysis. In conclusion, this study first investigated the chemical composition and potential pharmacological effects of the main chemical compounds in QZC, which will contribute to the revelation of bioactive compounds in QZC and provide evidence for clinical application.

## 1. Introduction

Rheumatoid arthritis (RA) is a chronic inflammatory joint disease of an autoimmune nature, featuring persistent inflammatory synovitis, whose progression is influenced by multiple factors including environmental, genetic, and infection factors [1–3]. With the accelerated pace of life and increased work pressure, the number of people suffering from RA in China is increasing annually, which is difficult to heal and prone to recurrence, seriously affecting their physical and mental health, and quality of life. Besides, nonsteroidal anti-inflammatory drugs and slow-acting antirheumatic drugs, mainly adopted by western medicine, usually lead to gastrointestinal side effects and psychological disorders. RA belongs to the category of “obstinate rheumatism” in traditional Chinese medicine (TCM), which is usually treated by removing wind and cold, dispelling dampness and clearing heat, eliminating blood stasis, and promoting blood circulation with remarkable efficacy [4], where Qufeng Zhitong capsule (QZC) is one of the effective drugs listed in the national essential medicine for the treatment of RA [5].

QZC, an effective patent medicine for treating RA, is composed of seven herbs that play different roles. Herba Geranii (Laoguancao, HG) and Radix Aconiti Kusnezoffii Cocta (Zhicaowu, RAKC) are monarch herbs that mainly exert the effect of dispelling wind and dampness, relaxing tendons, and relieving pain. Flos Carthami (Honghua, FC) and Radix Angelicae Pubescentis (Duhuo, RAP) serve as minister drugs with the function of dispelling wind and dredging collaterals, activating blood circulation and stimulating meridians, and reducing swelling and relieving pain. Radix Dipsaci (Xuduan, RD), Radix et Rhizoma Clematidis (Weilingxian, ReRC), and Herba Visci (Hujisheng, HV) all act as assistant drugs to possess the effects of dispelling wind and dampness, dredging collaterals and relieving pain, invigorating the liver and kidney, and strengthening tendons and bones [6]. With the integrated effects of herbs, QZC can be widely used to alleviate the symptoms of RA, such as limb numbness, waist and knee pain, owing to the synergism of these herbal medicines [7].

At present, most studies of QZC focus on its clinical applications with few analytical studies on its chemical compounds, causing poorly clarified chemical substances. In addition, little attention has been paid to the identification and quality evaluation of the overall medical product. QZC, as a TCM prescription, also has the characteristics of multicomponents, multitargets, and multipathways, and its comprehensive action mechanism on RA is still unclear. Li et al. reported that QZC has a positive effect on cortical bone and can prevent bone erosion in patients with RA [8]. With the rapid development of bioinformatics, network pharmacology, characterized by the holistic idea of TCM, has become a powerful tool and has been successfully applied to forecast the complicated action mechanism of TCM in the remedy of multiple illnesses by constructing and analyzing biological networks. Using existing databases, the potential mechanisms of Chinese herbs can be predicted, which can provide a theoretical reference for in-depth studies on the underlying mechanisms of TCM prescriptions [9].

In this study, a fast and effective method was developed for the chemical characterization of QZC using ultra-high performance liquid chromatography tandem Q-Exactive Orbitrap high-resolution mass spectrometry (UHPLC/Q-Orbitrap-MS). The systematic pharmacological mechanism of QZC in the treatment of RA was predicted via network pharmacology. This is the first study focusing on the analysis of the chemical types, herb origins, and potential pharmacological effects of the main chemical components in QZC, whose results can provide a reference for future research on the chemical compositions, pharmacological effects, and clinical applications of QZC.

## 2. Materials and Methods

### 2.1. Reagents and Materials

Methanol and formic acid were purchased from Fisher Scientific (Fair Lawn, NJ, USA). Dimethyl sulfoxide (DMSO) was acquired from Meridian Medical Technologies (MREDA, New York, NY, USA). Water for UHPLC/Q-Orbitrap-MS analysis was purified by the Milli-Q water purification system (Millipore, Billerica, MA, USA). QZC samples were provided by Shaanxi Buchang Pharmaceutical Co., Ltd. (Shaanxi, China). Reference compounds were obtained from Shanghai Yuanye Bio-Technology Co., Ltd. (Shanghai, China), including gallic acid, protocatechuic acid, neochlorogenic acid, chlorogenic acid, cryptochlorogenic acid, loganic acid, corilagin, loganin, isochlorogenic acids A−C, angelol A, columbianetin acetate, osthole, columbianadin, akebia saponin D, 5-hydroxymethyl furfural, 4-hydroxybenzoic acid, caffeic acid, hydroxysafflor yellow A, syringin, ferulic acid, secoxyloganin, ellagic acid, columbianetin, angelol G, and isoimperatorin. Benzoylmesaconine, benzoylaconine, and benzoylhypaconine were obtained from Shanghai Acmec Biochemical Co., Ltd. (Shanghai, China). Kaempferol-3-*O*-rutinoside was obtained from Chengdu Pufei De Biotech Co., Ltd. (Chengdu, China). The purities of these reference compounds were all determined to be above 98% by UPLC analysis.

### 2.2. Standard Solution Preparation

Thirty-one standards were accurately weighed and dissolved using methanol in volumetric flasks to obtain individual stock solutions. A certain amount of each stock solution was placed in a 10 mL volumetric flask and diluted to volume with 10% methanol aqueous solution for preparing a mixed reference solution at a final concentration of 0.0603 mg·mL^−1^ gallic acid, 0.0323 mg·mL^−1^ protocatechuic acid, 0.0714 mg·mL^−1^ neochlorogenic acid, 0.0451 mg·mL^−1^ chlorogenic acid, 0.0506 mg ·mL^−1^ cryptochlorogenic acid, 0.0603 mg·mL^−1^ loganic acid, 0.0537 mg·mL^−1^ corilagin, 0.0539 mg·mL^−1^ loganin, 0.0498 mg·mL^−1^ isochlorogenic acid B, 0.0499 mg·mL^−1^ isochlorogenic acid A, 0.0399 mg·mL^−1^ isochlorogenic acid C, 0.0543 mg·mL^−1^ angelol A, 0.0502 mg·mL^−1^ columbianetin acetate, 0.0494 mg·mL^−1^ osthole, 0.0320 mg·mL^−1^ columbianadin, 0.0467 mg·mL^−1^ akebia saponin D, 0.0500 mg·mL^−1^ 5-hydroxymethyl furfural, 0.0500 mg·mL^−1^ 4-hydroxybenzoic acid, 0.0501 mg·mL^−1^ caffeic acid, 0.0501 mg·mL^−1^ hydroxysafflor yellow A, 0.0500 mg·mL^−1^ syringin, 0.0505 mg·mL^−1^ ferulic acid, 0.0498 mg·mL^−1^ secoxyloganin, 0.0503 mg·mL^−1^ ellagic acid, 0.0506 mg·mL^−1^ benzoylmesaconine, 0.0500 mg·mL^−1^ kaempferol-3-*O*-rutinoside, 0.0505 mg·mL^−1^ benzoylaconine, 0.0504 mg mL^−1^ benzoylhypaconine, 0.0502 mg ·mL^−1^ columbianetin, 0.0498 mg·mL^−1^ angelol G, and 0.0498 mg·mL^−1^ isoimperatorin, respectively. All solutions were stored at 4°C when not in use.

#### 2.2.1. Sample Solution Preparation

The accurately weighed QZC powder (0.5 g) was transferred into a 25 mL volumetric flask and ultrasonically extracted with a certain amount of 75% methanol aqueous solution at 60°C for 30 min, which was subsequently diluted to scale by 75% methanol aqueous solution after cooling down to room temperature.

Weilingxian and Duhuo were pulverized into homogeneous powder, respectively. Then, 0.5 g was respectively weighed and ultrasonically extracted with 75% methanol aqueous solution (25 mL) at 60°C for 30 min. Honghua (10 g), Laoguancao (10 g), Hujisheng (10 g), Zhicaowu (10 g), and Xuduan (10 g) were respectively added to 80 mL of water and extracted with reflux extraction twice (3 h each time).

QZC, Weilingxian, and Duhuo sample solutions were diluted 25 times with water, while Honghua, Laoguancao, Hujisheng, Zhicaowu, and Xuduan sample solutions were diluted 25 times with 10% methanol aqueous solution for UHPLC/Q-Orbitrap-MS analysis, respectively.

### 2.3. UHPLC/Q-Orbitrap-MS Analysis

The qualitative analysis was performed on a UHPLC/Q-Orbitrap-MS (Thermo Fisher Scientific, San Jose, CA, USA) with chromatographic separation achieved using an ACQUITY UPLC® BEH C18 (2.1 × 100 mm, 1.7 *µ*m, Waters, Milford, MA, USA) at 40°C. The mobile phase system was composed of 0.1% formic acid aqueous solution (*v/v*) (*A*) and methanol (B) at 0.3 mL min^−1^, which was performed according to following optimized gradient program: 0–5 min, 3%–9% B; 5–9 min, 9%–15% B; 9–11 min, 15%–17% B; 11–15 min, 17%–27% B; 15–18 min, 27%–28% B; 18–26 min, 28%–50% B; 26–31 min, 50%–66% B; 31–35 min, 66%–74% B; and 35–36 min, 74%–3% B. The mass spectrometer was carried out in both positive and negative ion modes with centroided MS and MS^2^ spectra recorded from 100 to 1500 *m/z* in full MS and dd-MS^2^ (TopN) modes at a resolution of 70000 and 17500, respectively. The maximum injection time was set at 50 and 100 ms for MS^1^ and MS^2^, respectively. The automatic gain control (AGC) target of MS^1^ and MS^2^ was 3e^6^ and 1e^5^, respectively. The optimal MS parameters were as follows: spray voltage, −2.5 kV in negative ion mode and 3.5 kV in positive ion mode; sheath gas flow rate, 35 Arb; aux gas flow rate, 10 Arb; capillary temperature, 350°C; aux gas heater temperature, 350°C; collision energy at 20, 40, and 60 eV. All the data were acquired and processed by the Thermo Scientific™ Xcalibur™ system.

### 2.4. Network Pharmacology Analysis

The structural formulae and IUPAC-NIST chemical identifiers (InChIs) of the compounds were obtained from the PubChem database (https://pubchem.ncbi.nlm.nih.gov/), which were uploaded to the SwissTargetPrediction (https://www.swisstargetprediction.ch/) and BATMAN-TCM database (https://bionet.ncpsb.org.cn/batman-tcm/) to acquire their targets. All the targets were de-duplicated and then imported to Ingenuity Pathway Analysis (IPA) for core analysis so as to identify canonical pathways and networks of compounds, targets, and diseases.

## 3. Results and Discussion

### 3.1. Characterization of Chemical Constituents in Qufeng Zhitong Capsule by UHPLC/Q-Orbitrap-MS

**Figure 1:**
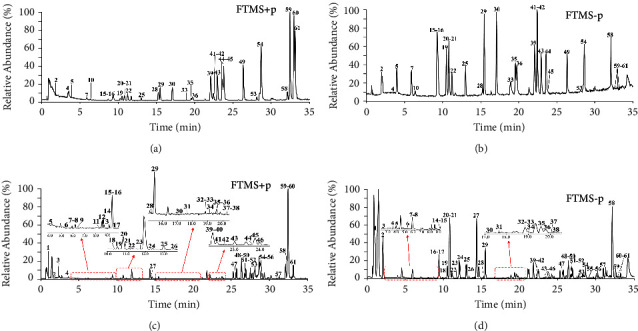
Total ion chromatograms of reference compounds in (a) positive ion mode and (b) negative ion mode and QZC in (c) positive ion mode and (d) negative ion mode.

**Figure 2:**
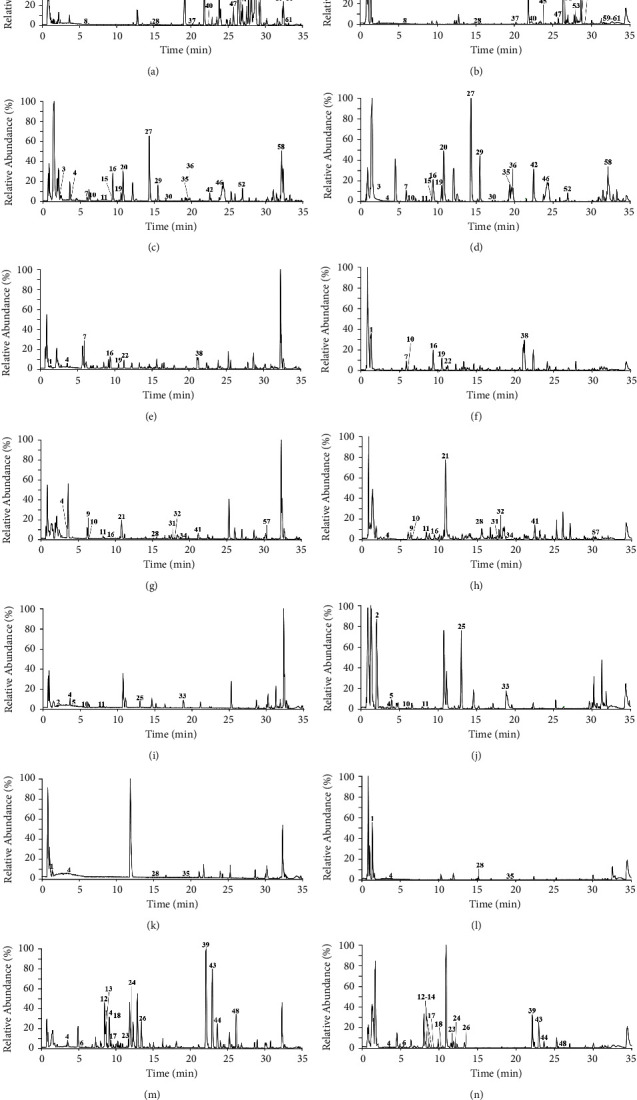
Total ion chromatograms of raw herbs from QZC. Duhuo extract in (a) positive ion mode and (b) negative ion mode, Xuduan extract in (c) positive ion mode and (d) negative ion mode, Hujisheng extract in (e) positive ion mode and (f) negative ion mode, Honghua extract in (g) positive ion mode and (h) negative ion mode, Laoguancao extract in (i) positive ion mode and (j) negative ion mode, Weilingxian extract in (k) positive ion mode and (l) negative ion mode, Zhicaowu extract in (m) positive ion mode and (n) negative ion mode.

The chemical compounds of QZC were identified by UHPLC/Q-Orbitrap-MS analysis in both positive and negative ion modes. As a result, 61 compounds were characterized by comparison with reported literatures, including 16 phenylpropanoids, 15 organic acids, 13 alkaloids, seven flavonoids, six iridoids, one saccharide, two aldehydes, and one saponin, among which 31 compounds were confirmed by comparison with the reference substances. The fragment ions of identified compounds are shown in Table 1, and the MS spectra of QZC sample and reference compounds are displayed in Figure 1, respectively. The MS spectra of individual herbs are shown in Figure 2. The chemical structures of the compounds identified by comparison with the reference substances in QZC are shown in Figure S1.

Coumarins, which are widely distributed in the plant families of Rutaceae and Umbelliferae, have been reported to have various therapeutic properties, such as antibacterial, antitumor, and antidepression activities [22]. Coumarins in QZC were found to mainly come from Duhuo. Ions at *m/z* 187.03911 [M+H]^+^ of compound 40 was observed in positive ion mode, whose product ions at *m/z* 159.04410 [M + H−CO]^+^, 131.04938 [M + H−CO−CO]^+^, 143.04921 [M + H−CO_2_]^+^, and 115.05457 [M + H−CO_2_−CO]^+^ were also observed. Compound 40 was identified as psoralen following the cleavage rule of coumarin, which is consistent with the reported literature [13]. Additionally, compounds 8, 37, 45, 47, 49, 50, 51, 53, 54, 55, 56, 59, 60, and 61 were identified as umbelliferone, nodakenetin, columbianetin, angelol D, angelol A, angelol B, angelol K, angelol G, columbianetin acetate, isoangenomalin, angenomalin, osthole, isoimperatorin, and columbianadin, respectively.

With the continuous development of natural product chemistry and the constant improvement of analytical techniques, organic acid components in TCM, such as chlorogenic acid and gallic acid, have been discovered to have strong activity, drawing increasing attention globally. Organic acids refer to acidic compounds containing the carboxylic group (−COOH), mostly distributed in herbs with a sour taste, and have been demonstrated to possess various pharmacological activities, including anti-inflammatory, antioxidation, and inhibition of platelet aggregation, which have important clinical application value in the prevention and treatment of cardiovascular diseases [23]. In negative ion mode, organic acids exhibited similar cleavage behaviours by the loss of CO_2_, H_2_O, or CO. Compounds 1, 2, 5, 7, 10, 15, 16, 19, 25, 28, 33, 35, 36, 42, and 57 were identified as quinic acid, gallic acid, protocatechuic acid, neochlorogenic acid, 4-hydroxybenzoic acid, caffeic acid, chlorogenic acid, cryptochlorogenic acid, corilagin, ferulic acid, ellagic acid, isochlorogenic acid B, isochlorogenic acid A, isochlorogenic acid C, and 9,12,13-trihydroxy-10-octadecenoic acid, respectively. Quinic acid is mainly present in Hujisheng. The mass spectrum of compound 1 in negative ion mode showed a quasimolecular ion at *m/z* 191.05511 [M−H]^−^ with its molecular formula speculated as C_7_H_12_O_6_, where its fragment ions were detected at *m/z* 173.00810 [M−H−H_2_O]^−^, 127.03873 [M−H−2H_2_O−CO]^−^, and 109.02816 [M−H−3H_2_O−CO]^−^. Compared with the cleavage pattern reported in the literature, compound 1 was confirmed as quinic acid [10]. Isochlorogenic acids A−C, neochlorogenic acid, chlorogenic acid, and cryptochlorogenic acid have been shown to contain quinic acid. Compound 33 was identified as ellagic acid, a common dimer of gallic acid, which was characterized by the presence of a quasimolecular ions at *m/z* 300.99890 [M−H]^−^ with fragment ion at *m/z* 257.00940 [M−H−CO_2_]^–^ and 229.01354 [M−H−CO_2_−CO]^–^ [19].

Iridoids, a type of monoterpene derivative in TCM, are widely distributed and have a variety of biological activities, such as antitumor, antiviral, and anti-inflammatory. Iridoids feature complex structures, mostly containing hemiacetal hydroxyl groups and cyclopentane rings, which are prone to the formation of iridoid glycosides with saccharides. The loss of monosaccharides or monosaccharide residues and neutral molecules such as CO_2_ (44), H_2_O (18), C_2_H_2_ (26), and CH_2_O (30), usually occurs in the mass spectra of iridoids [18]. The iridoids in QZC are mostly derived from XuDuan. In negative ion mode, compound 30 exhibited an ion with *m/z* value of 403.12555 [M−H]^−^, and produced fragment ions at *m/z* 223.06062 [M−H−Glc]^−^, 165.05470 [M−H−Glc−CH_2_COO]^−^, 121.02822 [M−H−Glc−CH_2_COO−CO_2_]^−^, and 95.04887 [M−H−Glc−CH_2_COO−CO_2_−C_2_H_2_]^−^, which are consistent with the mass spectrum fragmentation of secoxyloganin. According to the quasimolecular ions and the secondary fragment ions information, the fragmentation patterns of iridoid glycosides were analyzed. Compounds 20, 27, 29, 46, and 52 were identified as loganic acid, sweroside, loganin, cantleyoside, and triplostoside A.

Aconitine-type alkaloids are the primary active ingredients of Zhicaowu in QZC, which are both effective and toxic components with analgesic, anti-inflammatory, and circulation-promoting effects [24]. Zhicaowu has the effects of dispelling wind, removing dampness, warming menstruation, and relieving pain, which is related to its alkaloids, such as mesaconitine, hypaconitine, and karakoline. In total, 13 alkaloids from QZC were only detected in positive ion mode. Alkaloids with acetyl and benzoyl groups at C-8 and C-14, respectively, are called diester diterpenoid alkaloids (DDAs). Given that C-8 is the first active site of DDAs, DDAs tend to lose acetyl side chains so as to form stable fragment ions. AcOH (60) is usually removed from C-8 when the diester alkaloids are cleaved. Compound 48 showed quasimolecular ion at *m/z* 616.31207 [M+H]^+^ in positive ion mode with the product ions at *m/z* 556.29083 [M + H−AcOH]^+^, 524.26385 [M + H−AcOH−CH_3_OH]^+^, and 496.27423 [M + H−AcOH−CH_3_OH−CO]^+^, which was preliminary identified as hypaconitine [16]. Compounds 6, 12, 13, 14, 17, 18, 23, 24, 26, 39, 43, and 44 were identified as karakoline, songorine, mesaconine, isotalatizidine, napelline, aconine, hypaconine, neoline, talatisamine, benzoylmesaconine, benzoylaconine, and benzoylhypaconine, respectively.

Flavonoids are an essential class of active ingredients in TCM with a wide range of biological activities including vasodilation, antihepatic injury, antispasmodic, antibacterial, antiviral, antitumor, and antioxidant, which can also be used as food additives, natural antioxidants, natural pigments, and so on. The Retro-Diels-Alder (RDA) cleavage reaction involves the loss and rearrangement of flavonoid aglycone C ring in different ways in the positive ion mode, where [M+H]^+^ ions are subjected to collision-induced dissociation to lose neutral radicals or molecules, such as H_2_O, CO, and C_2_H_2_O_2_ [25], while flavonoid glycosides are prone to lose glycosyl during multistage cleavage. Flavonoids in QZC are mainly derived from Honghua. Compound 41 was tentatively identified as kaempferol-3-*O*-rutinoside due to the observation of quasimolecular ion at *m/z* 593.15094 [M–H]^–^in negative ion mode and the product ions at *m/z* 285.04004 [M−H−rutinose residue]^–^, 163.00266 [M−H−rutinose residue–C_7_H_6_O_2_]^–^, and 151.00261 [M−H−rutinose residue−C_8_H_6_O_2_]^–^. Compounds 9, 21, 31, 32, 34, and 38 were identified as safflomin A, hydroxysafflor yellow A, 6-hydroxykaempferol-3-*O*-glucoside, 6-hydroxykaempferol, carthamone, and homoeriodictyol-7-*O*-*β*-D-apiosyl-(1 ⟶ 2)-*β*-D-glucoside, respectively.

To further confirm the structures of the compounds, a total of 31 compounds were verified by comparison with reference standards, where compounds 2, 4, 5, 7, 10, 15, 16, 19−22, 25, 28, 29, 30, 33, 35, 36, 39, 41−45, 49, 53, 54, and 58−61 were unambiguously assigned as gallic acid, 5-hydroxymethylfurfural, protocatechuic acid, neochlorogenic acid, 4-hydroxybenzoic acid, caffeic acid, chlorogenic acid, cryptochlorogenic acid, loganic acid, hydroxysafflor yellow A, syringin, corilagin, ferulic acid, loganin, secoxyloganin, ellagic acid, isochlorogenic acid B, isochlorogenic acid A, benzoylmesaconine, kaempferol-3-*O*-rutinoside, isochlorogenic acid C, benzoylaconine, benzoylhypaconine, columbianetin, angelol A, angelol G, columbianetin acetate, akebia saponin D, osthole, isoimperatorin, and columbianadin, respectively (Table S1).

As an important ingredient in QZC, RAKC was reported to have alkaloids, such as aconine, mesaconine, and hypaconine, which were responsible for anti-inflammation and analgesia functions. Notably, toxicity would happen when RAKC was misused, which must be concerned about. The pharmacopoeia of the People's Republic of China stipulates that the total amount of aconitine, mesaconitine, and hypaconine must be no more than 0.04%, and the total amount of benzoylaconitine, benzoylhypaconine, and benzoylmesaconine must range from 0.02% to 0.07% [7]. Importantly, the dosage and the course of treatment of QZC must be paid more attention to in clinical use by taking the detected risky alkaloids from RAKC into account.

HG, RAP, RD, and FC are effective ingredients in QZC. From HG, corilagin was reported to exert antirheumatoid arthritis via downregulation of NF-*κ*B and MAPK signaling pathways [26]. The osthole in RAP can reduce proinflammatory cytokines, such as TNF-*α*, IL-1*β,* and IL-6, showing promising analgesic effects [27]. Hydroxysafflor yellow A, which derived from FC, can reduce expression of inflammatory mediators in synovial tissue, thus reducing inflammation and synovitis [28]. In addition, asperosaponin VI, one of the saponins in RD, can significantly inhibit RANKL-induced osteoclast formation to relieve arthritis *in vivo* [29]. The abovementioned compounds play an important role in the treatment of RA.

### 3.2. Network Pharmacology

As an open-source database, the SwissTargetPrediction is a web-based tool which can predict the targets of any bioactive small molecule as ligands [30]. IPA is a graphical interface bioinformatics software based on cloud computing, which can search for some important information, such as genes, proteins, diseases, and classical pathways [31].

**Figure 3:**
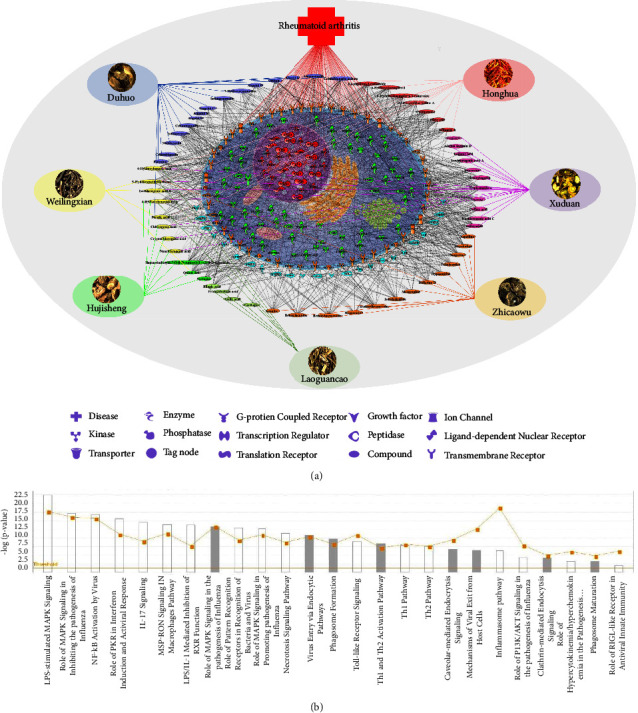
(a) The network model of “QZC-components-targets-RA” and (b) canonical pathway analysis for pathogen-influenced signaling of the predicted targets.

Based on the 61 compounds identified by UHPLC/Q-Orbitrap-MS, a network pharmacological analysis was carried out to preliminarily predict their potential pharmacological activities. The chemical structures and InChIs for 61 compounds from QZC were obtained from the PubChem database and literatures, which were uploaded to the SwissTargetPrediction database to obtain the targets of HOMO except for hypaconine, angelol A, angelol B, angelol D, angelol G, angelol K, columbianetin, mesaconine, and nodakenetin. The BATMAN-TCM database was used to predict the targets of the aforementioned nine compounds, with a cutoff score of 3.351. A total of 1002 targets were acquired after removing duplicates, which were imported to IPA software for core analysis with 926 targets detected. The 1002 targets corresponding to the 61 index compounds in QZC are shown in Table S2. RA was selected for analysis in the related diseases, resulting in 173 related targets, which are shown in Table S3. The network of “QZC-components-targets-RA” is shown in Figure 3(a). “Pathogen-influenced signaling” was selected for the reason that pathogen infection is an important problem in the research of RA [32]. The canonical pathway analysis for pathogen-influenced signaling of the predicted QZC targets is shown in Figure 3(b).

The pathogenesis of rheumatoid arthritis is still unclear. Current studies suggest that inflammation caused by an imbalance of Th1/Th2 cytokines is an important pathological mechanism [33]. Th1 cells mainly secrete proinflammatory cytokines, such as interferon-*γ* (IFN-*γ*) and interleukin-1 (IL-1), to promote the production of inflammatory cytokines. Th2 cells mainly secrete anti-inflammatory cytokines such as interleukin-4 (IL-4) and transforming growth factor-*β* (TGF-*β*), and inhibit the production of inflammatory cytokines [34]. Th1 and Th2 cells are balanced in healthy people, while Th1 cells increase and Th2 cells decrease in patients with RA [35]. NF-*κ*B, ICAM-1, JAK, IL-6, IL-23, CCR1, and CCR3 are closely associated with the Th1/Th2 signaling pathway, which may be regulated by the chemical compounds from QZC, suggesting that the chemical compounds from QZC may participate in the balance of the Th1/Th2 signaling pathway, thus, playing a therapeutic role in RA.

The results indicated that the identified compounds detected by UHPLC/Q-Orbitrap-MS have potential pharmacological activity for the treatment of RA, which sheds light on the modern approaches to investigate QZC and promoting the development of QZC.

## 4. Conclusions

In this study, 61 compounds in QZC were identified by UHPLC/Q-Orbitrap-MS, including 16 phenylpropanoids, 15 organic acids, 13 alkaloids, seven flavonoids, six iridoids, one saccharide, two aldehydes, and one saponin, with their herbal sources traced. Among them, 31 compounds were unambiguously identified by reference compounds, and another 30 compounds were tentatively characterized based on their fragmentation pathways. Network pharmacology analysis revealed that all detected compounds exhibited potential pharmacological activity in the treatment of RA via different signaling pathways. In conclusion, this is the first study focusing on the analysis of the overall chemical basis of QZC, which is conducive to revealing the bioactive compounds and potential application value of QZC.

## Figures and Tables

**Table 1:** Characterization of chemical constituents from Qufeng Zhitong capsule by UHPLC-Q Exactive Orbitrap MS.

Serial no.	t_R_/min	Formula	Theoretical value (m/*z*)	Measured value (m/*z*)	Error (ppm)	Fragment ions (m/*z*) (%)	Ion mode	Identification	Source
1	1.09	C_7_H_12_O_6_	191.05501	191.05511	0.52	191.05511 (100), 173.00810 (8.17), 127.03873 (4.84), 109.02816 (2.25)	−	Quinic acid [10]	HV, ReRC

2^*∗*^	1.97	C_7_H_6_O_5_	169.01357	169.01329	−1.66	169.01329 (35.95), 125.02315 (100)	−	Gallic acid	HG

3	2.31	C_12_H_22_O_11_	341.10894	341.10907	0.38	341.10892 (34.79), 180.06563 (100), 89.02310 (40.56), 59.01254 (82.79)	−	Sucrose [11]	RD

4^*∗*^	3.64	C_6_H_6_O_3_	127.03894	127.03912	1.42	127.03912 (22.83), 109.02877 (100), 81.03411 (3.51)	+	5-Hydroxymethylfurfural	RD, HG, FC, HV, RAKc, ReRC

5^*∗*^	3.93	C_7_H_6_O_4_	153.01878	153.01828	2.61	153.01828 (19.44) 109.02823 (100)	−	Protocatechuic acid	HG

6	5.39	C_22_H_35_NO_5_	394.25350	394.25864	−3.20	394.25864 (100), 376.24802 (67.05), 358.23752 (3.49)	+	Karakoline [12]	RAKc

7^*∗*^	5.84	C_16_H_18_O_9_	353.08671	353.08850	3.09	191.05533 (100), 179.03410 (55.29), 173.04460 (3.18), 161.02339 (3.81), 135.04395 (61.74)	−	Neochlorogenic acid	RD, HV

8	6.04	C_9_H_6_O_3_	163.03952	163.03889	−3.07	163.03889 (100), 145.02872 (35.33), 135.04420 (77.64)	+	Umbelliferone [13]	RAP

9	6.29	C_27_H_31_O_16_	611.16121	611.16107	−0.23	491.11966 (100)	−	Safflomin A [14]	FC

10^*∗*^	6.36	C_7_H_6_O_3_	137.02387	137.02462	5.47	137.02344 (16.81), 93.03324 (100)	−	4-Hydroxybenzoic acid	RD, FC, HG, HV

11	8.28	C_7_H_6_O_2_	123.04460	123.04431	−2.36	95.04965 (100)	+	4-Hydroxybenzaldehyde [15]	RD, FC, HG

12	8.47	C_22_H_31_NO_3_	358.23822	358.23776	−1.28	340.22733 (100), 58.06599 (5.78)	+	Songorine [12]	RAKc

13	8.69	C_24_H_39_NO_9_	486.27031	486.26990	−0.84	486.26990 (100), 436.23288 (10.55), 75.04474 (1.68)	+	Mesaconine [16]	RAKc

14	9.18	C_23_H_37_NO_5_	408.27500	408.27451	−1.20	390.26385 (31.65), 408.27451 (100)	+	Isotalatizidine [12]	RAKc

15^*∗*^	9.21	C_9_H_8_O_4_	179.03410	179.03409	−0.06	179.03409 (21.02), 135.04390 (100)	−	Caffeic acid	RD

16^*∗*^	9.32	C_16_H_18_O_9_	353.08789	353.08841	1.47	191.05531 (100), 179.03452 (0.67), 161.02342 (1.93), 135.04399 (1.09)	−	Chlorogenic acid	RD, FC, HV

17	9.34	C_22_H_33_NO_3_	360.25387	360.25336	−1.42	342.24283 (100)	+	Napelline [12]	RAKc

18	10.31	C_25_H_41_NO_9_	500.28596	500.28568	−0.56	450.24875 (8.62), 58.06598 (100)	+	Aconine [16]	RAKc

19^*∗*^	10.63	C_16_H_18_O_9_	353.08792	353.08762	−0.85	191.04462 (100), 179.03410 (64.34), 161.02251 (4.37), 135.04387 (69.27)	−	Cryptochlorogenic acid	RD, HV

20^*∗*^	10.74	C_16_H_24_O_10_	375.12976	375.12973	−0.08	213.07625 (100), 169.04604 (27.05), 151.07535 (11.38)	−	Loganic acid	RD

21^*∗*^	10.93	C_27_H_32_O_16_	613.17688	613.17639	−0.80	433.11356 (24.5), 415.10266 (12.10), 355.08136 (18.62), 211.02388 (100)	+	Hydroxysafflor yellow A	FC

22^*∗*^	11.19	C_17_H_24_O_9_	417.14124	417.14117	−0.17	209.08131 (37.15), 59.01241 (100)	−	Syringin	HV

23	11.77	C_24_H_39_NO_8_	470.27539	470.27505	−0.72	470.27505 (100), 438.24896 (21.47), 94.06562 (5.19)	+	Hypaconine [12]	RAKc

24	12.14	C_24_H_39_NO_6_	438.28556	438.28540	−0.37	438.28540 (100), 388.24857 (3.00), 356.22232 (1.41)	+	Neoline [12]	RAKc

25^*∗*^	12.92	C_27_H_22_O_18_	633.07294	633.07214	−1.26	300.99875 (100), 257.00870 (7.06), 229.01340 (7.48)	−	Corilagin	HG

26	13.53	C_24_H_39_NO_5_	422.29065	422.29022	−1.02	422.29022 (100), 390.26398 (28.76)	+	Talatisamine [12]	RAKc

27	14.41	C_16_H_22_O_9_	403.12404	403.12534	3.23	357.11902 (13.66), 195.06557 (20.18), 125.02322 (100)	−	Sweroside [17]	RD

28^*∗*^	15.25	C_10_H_10_O_4_	195.06540	195.06530	−0.51	177.0547 (90.45), 149.96118 (36.76), 145.02849 (64.36), 117.03381 (32.89)	+	Ferulic acid	FC, ReRC, RAP

29^*∗*^	15.42	C_18_H_28_O_12_	435.15158	435.15176	0.41	227.09209 (100), 127.03885 (56.73), 101.02310 (60.66)	−	Loganin	RD

30^*∗*^	17.12	C_17_H_24_O_11_	403.12521	403.12555	0.84	223.06062 (18.87), 165.05470 (20.64), 121.02822 (100), 95.04887 (35.91)	−	Secoxyloganin [18]	RD

31	17.64	C_21_H_20_O_12_	463.08765	463.08911	3.15	301.03543 (100)	−	6-Hydroxykaempferol 3-*O*-glucoside or its isomer [14]	FC

32	18.82	C_15_H_10_O_7_	303.05048	303.04993	−1.82	303.04993 (100), 285.03894 (3.51), 257.04492 (5.75), 229.04944 (12.61)	+	6-Hydroxykaempferol or its isomer [14]	FC

33^*∗*^	18.82	C_14_H_6_O_8_	300.99844	300.99890	1.53	300.99890 (100), 257.00940 (2.70), 229.01353 (4.23), 283.99612 (4.92), 201.01845 (3.67)	−	Ellagic acid [19]	HG

34	19.1	C_21_H_20_O_11_	447.09274	447.09311	0.83	285.0401 (100)	−	Carthamone [14]	FC

35^*∗*^	19.47	C_25_H_24_O_12_	515.11938	515.11908	−0.58	535.08786 (59.70), 191.05531 (50.97), 173.04468 (100)	−	Isochlorogenic acid B	RD, ReRC

36^*∗*^	19.91	C_25_H_24_O_12_	515.11920	515.11902	−0.35	535.08771 (57.67), 191.05530 (100), 173.04460 (11.88)	−	Isochlorogenic acid A	RD

37	20.16	C_14_H_14_O_4_	247.09703	247.09663	−1.62	247.09663 (100), 229.08597 (28.87), 201.09113 (1.50), 147.04411 (6.53), 131.04933 (19.54), 119.04932 (2.93)	+	Nodakenetin [13]	RAP

38	20.22	C_27_H_32_O_15_	595.16630	595.16547	−1.39	301.07178 (88.62), 151.00261 (100), 107.01261 (20.31), 65.00.190 (10.98)	−	Homoeriodictyol-7-*O*-*β*-D-apiosyl-(1 ⟶ 2)-*β*-D-glucoside [20]	HV

39^*∗*^	22.21	C_31_H_43_NO_10_	590.29626	590.29553	−1.24	590.29553 (100), 540.25885 (9.31), 105.03378 (77.85)	+	Benzoylmesaconine	RAKc

40	22.29	C_11_H_6_O_3_	187.03952	187.03911	−2.19	187.03911 (100), 159.0441 (2.87), 143.04921 (13.44), 131.04938 (42.93), 115.05457 (28.41)	+	Psoralen [13]	RAP

41^*∗*^	22.4	C_27_H_30_O_15_	593.15065	593.15094	1.42	285.04004 (100), 163.00266 (2.98), 151.00261 (3.61)	−	Kaempferol-3-*O*-rutinoside	FC

42^*∗*^	22.6	C_25_H_24_O_12_	515.12067	515.11896	−3.32	535.08777 (72.08), 191.05525 (42.04), 173.04459 (100)	−	Isochlorogenic acid C	RD

43^*∗*^	23.03	C_32_H_45_NO_10_	604.31189	604.31183	−0.10	604.31183 (100), 554.24884 (8.61), 105.03386 (62.41)	+	Benzoylaconine	RAKc

44^*∗*^	23.67	C_31_H_43_NO_9_	574.30121	574.30127	0.10	574.30127 (100), 542.27515 (19.52), 105.03387 (66.9)	+	Benzoylhypaconine	RAKc

45^*∗*^	23.88	C_14_H_14_O_4_	247.09668	247.09654	−0.57	247.09654 (82.2), 229.08595 (12.87), 201.05467 (4.15), 175.03903 (100)	+	Columbianetin	RAP

46	23.95	C_33_H_46_O_19_	791.26098	791.26380	3.56	513.16101 (82.82), 459.15118 (44.71), 143.04921 (13.44), 141.01816 (62.97), 59.01250 (100)	−	Cantleyoside [17]	RD

47	25.64	C_20_H_24_O_7_	377.16003	377.15967	−0.95	377.15967 (100), 277.10672 (2.85), 219.06523 (4.95), 205.04964 (23.52), 191.03392 (62.76), 175.03906 (22.23), 160.05196 (9.69), 147.04404 (3.10), 131.04927 (3.20)	+	Angelol D [13]	RAP

48	26.22	C_33_H_45_NO_10_	616.31217	616.31207	−0.16	616.31207 (100), 556.29083 (15.79), 524.26385 (15.08), 496.27423 (4.06)	+	Hypaconitine [16]	RAKc

49^*∗*^	26.3	C_20_H_24_O_7_	377.15942	377.15918	−0.64	377.15918 (100), 259.09644 (3.78), 219.06508 (6.77), 191.03375 (62.86)	+	Angelol A	RAP

50	26.47	C_20_H_24_O_7_	377.16003	377.15967	−0.95	377.15967 (100), 277.10672 (3.01), 219.06508 (6.52), 205.04944 (24.79), 191.03374 (60.47), 175.03891 (24.15), 160.05177 (9.98), 147.04391 (2.89), 131.04916 (3.53)	+	Angelol B [13]	RAP

51	26.66	C_20_H_24_O_7_	377.16003	377.15967	−0.95	377.15967 (100), 277.10654 (2.78), 219.06528 (6.67), 205.04968 (23.38), 191.03395 (58.86), 175.03902 (22.27), 160.05199 (9.83), 147.04385 (2.88), 131.04900 (3.03)	+	Angelol K [13]	RAP

52	26.72	C_35_H_52_O_20_	837.30285	837.30530	2.93	629.24457 (53.41), 495.15112 (85.67), 459.15082 (47.06), 419.15601 (29.72), 209.08188 (21.86), 113.02312 (15.89), 101.02306 (100)	−	Triplostoside A [17]	RD

53^*∗*^	28.17	C_20_H_24_O_7_	377.15955	377.15970	0.40	377.15970 (100), 219.06535 (84.13), 205.04908 (37.19), 191.03407 (23.93)	+	Angelol G	RAP

54^*∗*^	28.75	C_16_H_16_O_5_	289.10754	289.10638	−4.01	229.08571 (45.08), 187.03883 (100), 175.03886 (15.25), 159.04395 (22.05), 147.0439 (2.92), 143.04909 (3.35), 131.04912 (24.23)	+	Columbianetin acetate	RAP

55	29.01	C_14_H_12_O_3_	229.08647	229.08598	−2.14	229.08598 (100), 201.12773 (5.25), 187.03900 (72.70), 159.04416 (30.70), 147.04401 (7.49), 131.04930 (36.47)	+	Isoangenomalin [13]	RAP

56	29.09	C_14_H_12_O_3_	229.08647	229.08624	−1.00	229.08624 (100), 201.16721 (27.01), 187.03914 (67.53), 159.04413 (25.83), 147.04410 (7.64), 131.04939 (21.57)	+	Angenomalin [13]	RAP

57	30.98	C_18_H_34_O_5_	329.23280	329.23343	1.91	329.23343 (100), 311.22229 (3.08), 171.10187 (13.07)	−	9,12,13-Trihydroxy-10-octadecenoic acid [21]	FC

58^*∗*^	32.22	C_47_H_76_O_18_	973.50385	973.50336	−0.50	927.49628 (7.91), 603.39175 (100), 323.09830 (15.04), 179.05513 (3.77)	−	Akebia saponin D	RD

59^*∗*^	32.64	C_15_H_16_O_3_	245.11739	245.11714	−1.02	189.05455 (100), 161.05965 (4.94), 159.04401 (6.16)	+	Osthole	RAP

60^*∗*^	32.87	C_16_H_14_O_4_	271.09644	271.09641	−0.11	203.03378 (100), 175.03896 (2.75), 159.04402 (9.99), 147.04399 (31.76), 131.04931 (11.46), 119.04935 (2.77)	+	Isoimperatorin	RAP

61^*∗*^	33.08	C_19_H_20_O_5_	329.13828	329.13821	−0.21	229.08594 (80.17), 201.05467 (2.97), 187.03899 (100), 175.03902 (23.19), 173.05981 (3.99), 159.04408 (28.31), 147.04413 (3.70), 143.04915 (3.52), 131.04924 (29.65)	+	Columbianadin	RAP

^*∗*^Compared with the reference standard.

## Data Availability

The data used to support the findings of this study are included within the article and the supplementary information file.

## Supplementary Materials

Supplementary Materials Figure S1: chemical structures of the compounds identified by comparison with reference substances. Table S1: characterization of chemical constituents from QZC by comparison with the reference substances. Table S2: 1002 targets corresponding to the 61 index compounds in QZC. Table S3: 173 targets related to rheumatoid arthritis.

## References

[1] Smolen J. S., Aletaha D., Barton A. (2018). Rheumatoid arthritis. *Nature Reviews Disease Primers*.

[2] Mcinnes I. B., Schett G. (2017). Pathogenetic insights from the treatment of rheumatoid arthritis. *The Lancet*.

[3] Kronzer V. L., Westerlind H., Alfredsson L. (2020). Respiratory diseases as risk factors for seropositive and seronegative rheumatoid arthritis and in relation to smoking. *Arthritis & Rheumatology*.

[4] Lu M. C., Livneh H., Chiu L. M., Lai N. S., Yeh C. C., Tsai T. Y. (2019). A survey of traditional Chinese medicine use among rheumatoid arthritis patients: a claims data-based cohort study. *Clinical Rheumatology*.

[5] Li X. Q., Jia C. Y., Xin N. (2010). Clinical analysis of Qufeng Zhitong capsule in the treatment of rheumatoid arthritis. *China Practical Medical*.

[6] Li Y. J. (2017). Analysis of clinical efficacy of dispelling analgesic capsule in rheumatoid arthritis. *Inner Mongolia Journal of Traditional Chinese Medicine*.

[7] Editorial Committee of Chinese Pharmacopoeia (2020). *Chinese Pharmacopoeia*.

[8] Li L., Yi X. M., Huang C. S. (2020). Qu Feng Zhi Tong capsule increases mechanical properties of cortical bone in ovariectomised rats. *Journal of Orthopaedic Translation*.

[9] Guo Q., Zheng K., Fan D. (2017). Wu-Tou Decoction in rheumatoid arthritis: integrating network pharmacology and *in vivo* pharmacological evaluation. *Frontiers in Pharmacology*.

[10] Gan C., Liu L., Du Y. (2016). Simultaneous determination and pharmacokinetic study of four phenol compounds in rat plasma by ultra-high performance liquid chromatography with tandem mass spectrometry after oral administration of *Echinacea purpurea* extract. *Journal of Separation Science*.

[11] Gabbanini S., Lucchi E., Guidugli F., Matera R., Valgimigli L. (2010). Anomeric discrimination and rapid analysis of underivatized lactose, maltose, and sucrose in vegetable matrices by U-HPLC-ESI-MS/MS using porous graphitic carbon. *Journal of Mass Spectrometry*.

[12] Zhi M. R., Gu X. R., Han S. (2020). Chemical variation in aconti kusnezoffii Radix before and after processing based on UPLC-orbitrap-MS. *China Journal of Chinese Materia Medica*.

[13] Wan M., Zhang Y., Yang Y., Liu X., Jia L., Yang X. (2019). Analysis of the chemical composition of Angelicae Pubescentis Radix by ultra-performance liquid chromatography and quadrupole time-of-flight tandem mass spectrometry. *Journal of Chinese Pharmaceutical Sciences*.

[14] Wang S. S., Ma Y., Zhang Y., Li D. F., Yang H. J., Liang R. X. (2015). Rapid identification of chemical composition in safflower with UHPLC-LTQ-Orbitrap. *China Journal of Chinese Materia Medica*.

[15] Zhang X., Gao H., Wang N., Yao X. (2006). Phenolic components from *Dendrobium nobile*. *Chinese Traditional and Herbal Drugs*.

[16] Wang Y., Song F., Xu Q., Liu Z., Liu S. (2003). Characterization of aconitine-type alkaloids in the flowers of Aconitum kusnezoffii by electrospray ionization tandem mass spectrometry. *Journal of Mass Spectrometry*.

[17] Sun X., Zhang Y., Yang Y. (2018). Qualitative and quantitative analysis of furofuran lignans, iridoid glycosides, and phenolic acids in Radix Dipsaci by UHPLC-Q-TOF/MS and UHPLC-PDA. *Journal of Pharmaceutical and Biomedical Analysis*.

[18] Kucharska A. Z., Sokół-Łętowska A., Oszmianski J., Piórecki N., Fecka I. (2017). Iridoids, phenolic compounds and antioxidant activity of edible honeysuckle berries (*Lonicera caerulea* var. *kamtschatica* Sevast.). *Molecules*.

[19] Yan L., Yin P., Ma C., Liu Y. (2014). Method development and validation for pharmacokinetic and tissue distributions of ellagic acid using ultrahigh performance liquid chromatography-tandem mass spectrometry (UPLC-MS/MS). *Molecules*.

[20] Zhao Y., Yu Z., Fan R. (2011). Simultaneous determination of ten flavonoids from *Viscum coloratum* grown on different Host species and different sources by LC-MS. *Chemical & Pharmaceutical Bulletin*.

[21] Geng P., Harnly J. M., Chen P. (2015). Differentiation of whole grain from refined wheat (*T. aestivum*) flour using lipid profile of wheat bran, germ, and endosperm with UHPLC-HRAM mass spectrometry. *Journal of Agricultural and Food Chemistry*.

[22] Bhattarai N., Kumbhar A. A., Pokharel Y. R., Yadav P. N. (2021). Anticancer potential of coumarin and its derivatives. *Mini-Reviews in Medicinal Chemistry*.

[23] Li D., Zhou L., Wang Q., He Y. (2018). Determination of organic acids for quality evaluation in *Coptis* herbs by ion chromatography. *3 Biotech*.

[24] Jeon S. Y., Jeong W., Park J. S. (2021). Clinical relationship between blood concentration and clinical symptoms in aconitine intoxication. *The American Journal of Emergency Medicine*.

[25] Fang S., Qu Q., Zheng Y. (2016). Structural characterization and identification of flavonoid aglycones in three *Glycyrrhiza* species by liquid chromatography with photodiode array detection and quadrupole time-of-flight mass spectrometry. *Journal of Separation Science*.

[26] Shen Y., Teng L., Qu Y. (2022). Anti-proliferation and anti-inflammation effects of corilagin in rheumatoid arthritis by downregulating NF-*κ*B and MAPK signaling pathways. *Journal of Ethnopharmacology*.

[27] Li R., Zhao C., Yao M., Song Y., Wu Y., Wen A. (2017). Analgesic effect of coumarins from Radix angelicae pubescentis is mediated by inflammatory factors and TRPV1 in a spared nerve injury model of neuropathic pain. *Journal of Ethnopharmacology*.

[28] Li D. W., Wang X. T., Mu B. C., Dou D. Q., Kang T. G. (2021). Effects of hydroxysafflor yellow A on rats with collagen-induced arthritis. *Biochemical and Biophysical Research Communications*.

[29] Liu K., Liu Y., Xu Y. (2019). Asperosaponin VI protects against bone destructions in collagen induced arthritis by inhibiting osteoclastogenesis. *Phytomedicine*.

[30] Daina A., Michielin O., Zoete V. (2019). SwissTargetPrediction: updated data and new features for efficient prediction of protein targets of small molecules. *Nucleic Acids Research*.

[31] Chen C., Cui S., Li W. (2020). Ingenuity pathway analysis of human facet joint tissues: insight into facet joint osteoarthritis. *Experimental and Therapeutic Medicine*.

[32] Arleevskaya M. I., Kravtsova O. A., Lemerle J., Renaudineau Y., Tsibulkin A. P. (2016). How rheumatoid arthritis can result from provocation of the immune system by microorganisms and viruses. *Frontiers in Microbiology*.

[33] Li Y., Jie Y., Wang X., Lu J. (2021). Serum IL-35 is decreased in overweight patients with rheumatoid arthritis: its correlation with Th1/Th2/Th17-related cytokines. *BMC Immunology*.

[34] Niu Y., Dong Q., Li R. (2017). Matrine regulates Th1/Th2 cytokine responses in rheumatoid arthritis by attenuating the NF-*κ*B signaling. *Cell Biology International*.

[35] Zhou X., Hua X., Ding X., Bian Y., Wang X. (2011). Trichostatin differentially regulates Th1 and Th2 responses and alleviates rheumatoid arthritis in mice. *Journal of Clinical Immunology*.

