# Supplementary material for: Illumination on Chemical Compounds from Qufeng Zhitong Capsule and Its Potential Pharmacological Mechanism against Rheumatoid Arthritis Based on UHPLC/Q-Orbitrap-MS Combined with Network Pharmacology Analysis
Source: Int J Anal Chem. 2022 Dec 7;2022:7863435. doi: 10.1155/2022/7863435 (PMC9750772; doi:10.1155/2022/7863435)
Supplement: Supplementary Materials — Figure S1: chemical structures of the compounds identified by comparison with reference substances. Table S1: characterization of chemical constituents from QZC by comparison with the reference substances. Table S2: 1002 targets corresponding to the 61 index compounds in QZC. Table S3: 173 targets related to rheumatoid arthritis. [file 7863435.f1.docx]

**Supplementary Materials to:**

**Illumination on Chemical Compounds from Qufeng Zhitong Capsule and Its Potential Pharmacological Mechanism against Rheumatoid Arthritis Based on UHPLC/Q-Orbitrap-MS Combined with Network Pharmacology Analysis**


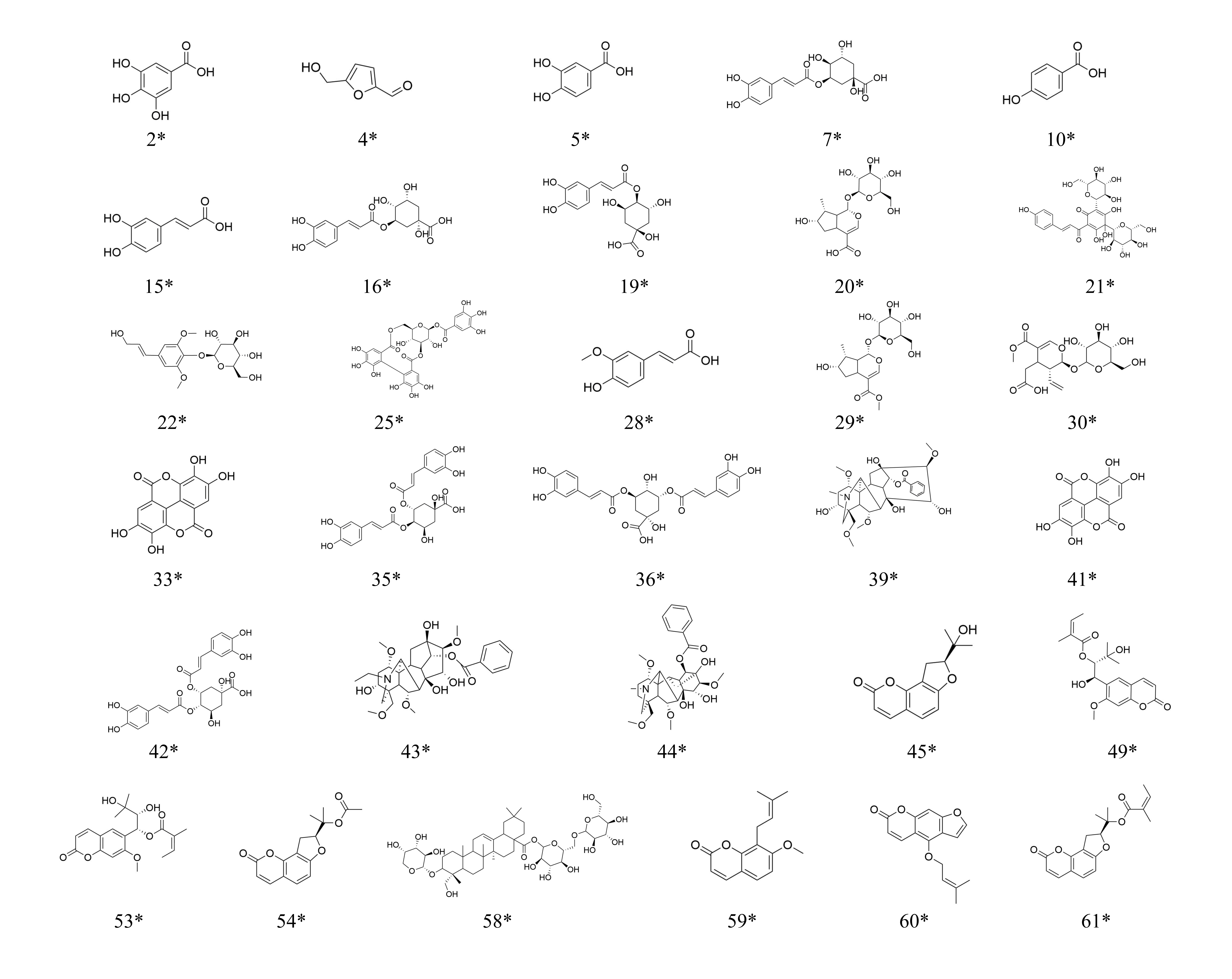


Figure S1: Chemical structures of the compounds identified by comparison with reference substances.

Table S1: Characterization of chemical constituents from QZC by comparison with the reference substances.

|  | | Detected compounds from QZC | | |  | Reference compounds from QZC | | | |  | |
| --- | --- | --- | --- | --- | --- | --- | --- | --- | --- | --- | --- |
| No. | Formula | *t_R_*/min | Measured  value (*m/z*) | Fragment ions (*m/z*) (%) |  | *t_R_*/min | Theoretical  value (*m/z)* | Fragment ions (*m/z*) (%) | Ion  mode | | Identification |
| 2 | C_7_H_6_O_5_ | 1.97 | 169.01329 | 169.01329 (35.95)  125.02315 (100) |  | 1.94 | 169.01335 | 169.01335 (38.18)  125.02317 (100) | – | | gallic acid |
| 4 | C_6_H_6_O_3_ | 3.64 | 127.03912 | 127.03912 (22.83)  109.02877 (100)  81.03411 (3.51) |  | 3.58 | 127.03894 | 127.03894 (6.98)  109.02856 (10.00)  81.03397 (47.98) | + | | 5-hydroxymethylfurfural |
| 5 | C_7_H_6_O_4_ | 3.93 | 153.01828 | 153.01828 (19.44)  109.02823 (100) |  | 3.86 | 153.01814 | 153.01814 (20.03)  109.02817 (100) | – | | protocatechuic acid |
| 7 | C_16_H_18_O_9_ | 5.84 | 353.08850 | 191.05533 (100)  179.03410 (55.29)  173.04460 (3.18)  161.02339 (3.81)  135.04395 (61.74) |  | 5.80 | 353.08789 | 191.05540 (100)  179.03421 (55.44)  173.04471 (3.33)  161.02344 (4.12)  135.04395 (68.50) | – | | neochlorogenic acid |
| 10 | C_7_H_6_O_3_ | 6.36 | 137.02462 | 137.02344 (16.81)  93.03324 (100) |  | 6.29 | 137.02322 | 137.02322 (11.80)  93.03323 (100) | – | | 4-hydroxybenzoic acid |
| 15 | C_9_H_8_O_4_ | 9.21 | 179.03409 | 179.03409 (21.02)  135.04390 (100) |  | 9.24 | 179.03405 | 179.03445 (24.77)  135.04405 (100) | – | | caffeic acid |
| 16 | C_16_H_18_O_9_ | 9.32 | 353.08841 | 191.05531 (100)  179.03452 (0.67)  161.02342 (1.93)  135.04399 (1.09) |  | 9.40 | 353.08789 | 191.05540 (100)  179.03421 (55.40)  161.02344 (4.12)  135.04395 (68.50) | – | | chlorogenic acid |
| 19 | C_16_H_18_O_9_ | 10.63 | 353.08762 | 191.04462 (100)  179.03410 (64.34)  135.04387 (69.27)  161.02251 (4.37) |  | 10.50 | 353.08844 | 191.05527 (100)  179.03424 (49.58)  135.04413 (43.56) | – | | cryptochlorogenic acid |
| 20 | C_16_H_24_O_10_ | 10.74 | 375.12973 | 213.07625 (100)  169.04604 (27.05)  151.07535 (11.38) |  | 10.80 | 375.12976 | 213.07632 (100)  169.08615 (28.17)  151.075425 (11.37) | – | | loganic acid |
| 21 | C_27_H_32_O_16_ | 11.03 | 613.17639 | 433.11356 (24.5),  415.10266 (12.10),  355.08136 (18.62),  211.02388 (100) |  | 10.83 | 613.17670 | 433.11316 (25.19)  415.10226 (11.34)  355.08130 (18.44)  211.02380 (100) | + | | hydroxysafflor yellow A |
| 22 | C_17_H_24_O_9_ | 11.19 | 417.14117 | 209.08131 (37.15),  59.01241 (100) |  | 11.22 | 417.14117 | 209.08138 (100)  59.01228 (0.69) | – | | syringin |
| 25 | C_27_H_22_O_18_ | 12.92 | 633.07214 | 300.99875 (100)  257.00870 (7.06)  229.01340 (7.48) |  | 12.90 | 633.07452 | 300.99896 (100)  257.00867 (6.67)  229.01297 (7.19) | – | | corilagin |
| 28 | C_10_H_10_O_4_ | 15.25 | 195.06530 | 177.05470 (90.45)  149.96118 (36.76)  145.02849 (64.36)  117.03381 (32.89) |  | 15.31 | 195.06534 | 177.05446 (100)  149.05960 (24.84)  145.02829 (78.35)  117.03365 (53.59) | + | | ferulic acid |
| 29 | C_18_H_28_O_12_ | 15.42 | 435.15176 | 227.09209 (100)  127.03885 (56.73)  101.02310 (60.66) |  | 15.48 | 435.15158 | 227.09207 (100)  127.03878 (53.70)  101.02308 (61.54) | – | | loganin |
| 30 | C_17_H_24_O_11_ | 17.12 | 403.12555 | 223.06062 (18.87)  165.05470 (20.64)  121.02822 (100)  95.04887 (35.91) |  | 17.09 | 403.12521 | 223.06046 (19.28)  165.05431 (23.05)  121.02811 (100)  95.04863 (21.69) | – | | secoxyloganin |
| 33 | C_14_H_6_O_8_ | 18.82 | 300.99890 | 300.99890 (100)  257.00940 (2.70)  229.01353 (4.23)  283.99612 (4.92)  201.01845 (3.67) |  | 18.77 | 300.99875 | 300.99875 (100)  257.00705 (2.31)  229.01364 (5.64)  283.99591 (4.54)  201.01765 (3.84) | – | | ellagic acid |
| 35 | C_25_H_24_O_12_ | 19.47 | 515.11908 | 535.08786 (59.70)  191.05531 (50.97)  173.04468 (100) |  | 19.58 | 515.12030 | 191.05530 (87.29)  173.04423 (100) | – | | isochlorogenic acid B |
| 36 | C_25_H_24_O_12_ | 19.91 | 515.11902 | 535.08771 (57.67)  191.05530 (100)  173.04460 (11.88) |  | 19.80 | 515.12030 | 191.05525 (100)  173.04475 (32.87) | – | | isochlorogenic acid A |
| 39 | C_31_H_43_NO_10_ | 22.21 | 590.29553 | 590.29553 (100)  540.25885 (9.31)  105.03378 (77.85) |  | 22.02 | 590.29510 | 590.29510 (100)  540.25842 (9.29)  105.03373 (96.65) | + | | benzoylmesaconine |
| 41 | C_27_H_30_O_15_ | 22.40 | 593.15094 | 285.04004 (100)  163.00266 (2.98)  151.00261 (3.61) |  | 22.32 | 593.15204 | 285.04010 (100)  163.00246 (2.36)  151.00266 (3.63) | – | | kaempferol-3-*O*-rutinoside |
| 42 | C_25_H_24_O_12_ | 22.60 | 515.11896 | 535.08777 (72.08)  191.05525 (42.04)  173.04459 (100) |  | 22.46 | 515.12012 | 191.05530 (87.29)  173.04463 (100) | – | | isochlorogenic acid C |
| 43 | C_32_H_45_NO_10_ | 23.03 | 604.31183 | 604.31183 (100)  554.24884 (8.61)  105.03386 (62.41) |  | 22.86 | 604.31189 | 604.31189 (100)  554.27502 (9.70)  105.043387 (77.65) | + | | benzoylaconine |
| 44 | C_31_H_43_NO_9_ | 23.67 | 574.30127 | 574.30127 (100)  542.27515 (19.52)  105.03387 (66.9) |  | 23.53 | 574.30029 | 574.30029 (100)  542.27417 (22.07)  105.03372 (91.30) | + | | benzoylhypaconine |
| 45 | C_14_H_14_O_4_ | 23.88 | 247.09654 | 247.09654 (82.2)  229.08595 (12.87)  201.05467 (4.15)  175.03903 (100) |  | 24.03 | 247.09634 | 247.09634 (81.54)  229.08583 (9.36)  201.05458 (4.00)  175.03889 (100) | + | | columbianetin |
| 49 | C_20_H_24_O_7_ | 26.30 | 377.15918 | 377.15918 (100)  259.09644 (3.78)  219.06508 (6.77)  191.03375 (62.86) |  | 26.50 | 377.15924 | 377.15924 (100)  259.09631 (4.00)  219.06505 (6.82)  191.03371 (58.09) | + | | angelol A |
| 53 | C_20_H_24_O_7_ | 28.17 | 377.15970 | 377.15970 (100)  219.06535 (84.13)  205.04908 (37.19)  191.03407 (23.93) |  | 28.04 | 377.15952 | 377.15952 (100)  219.06522 (34.26)  205.04962 (27.83)  191.03386 (44.06) | + | | angelol G |
| 54 | C_16_H_16_O_5_ | 28.75 | 289.10638 | 229.08571 (45.08)  187.03883 (100)  175.03886 (15.25)  159.04395 (22.05)  147.0439 (2.92)  143.04909 (3.35)  131.04912 (24.23) |  | 28.41 | 289.10666 | 229.08603 (50.58)  187.03906 (100)  175.03962 (17.92)  159.04419 (28.95)  147.04384 (2.82)  143.04926 (2.88)  131.04929 (22.06) | + | | columbianetin acetate |
| 58 | C_47_H_76_O_18_ | 32.22 | 973.50336 | 927.49628 (7.91)  603.39175 (100)  323.09830 (15.04)  179.05513 (3.77) |  | 32.42 | 973.50391 | 927.49115 (13.85)  603.39001 (100)  323.09827 (10.92)  179.05426 (3.70) | – | | akebia saponin D |
| 59 | C_15_H_16_O_3_ | 32.64 | 245.11714 | 189.05455 (100)  161.05965 (4.94)  159.04401 (6.16) |  | 32.36 | 245.11662 | 189.05423 (100)  161.05942 (5.15)  159.04373 (6.94) | + | | osthole |
| 60 | C_16_H_14_O_4_ | 32.87 | 271.09641 | 203.03378 (100)  175.03896 (2.75)  159.04402 (9.99)  147.04399 (31.76)  131.04931 (11.46)  119.04935 (2.77) |  | 32.87 | 271.09644 | 203.03384 (100)  175.03897 2.65)  159.04408 (10.74)  147.04404 (34.66)  131.04924 (13.12)  119.04939 (3.40) | + | | isoimperatorin |
| 61 | C_19_H_20_O_5_ | 33.08 | 329.13821 | 229.08594 (80.17)  201.05467 (2.97)  187.03899 (100)  175.03902 (23.19)  173.05981 (3.99)  159.04408 (28.31)  147.04413 (3.70)  143.04915 (3.52)  131.04924 (29.65) |  | 33.16 | 329.13840 | 229.08568 (80.69)  201.05446 (3.62)  187.03882 (100)  175.03885 (24.87)  173.05963 (3.96)  159.04393 (29.20)  147.04391 (3.90)  143.04909 (3.92)  131.04910 (28.94) | + | | columbianadin |

Table S2: 1002 targets corresponding to the 61 index compounds in QZC.

| Targets | | | | | | | | | | |
| --- | --- | --- | --- | --- | --- | --- | --- | --- | --- | --- |
| CHRNA7 | RPS6KA3 | GSTA1 | APP | ATP6AP1 | HTR2A | EIF2AK3 | BACE2 | CXCR2 | CASP7 | CREBBP |
| VEGFA | KCNMA1 | IDO1 | PYGL | P2RY4 | CES2 | MDM2 | CDK4 | CNR2 | FBP1 | CHRNA4 |
| FGF1 | GABRA2 | BCL2L1 | LGALS7 | P2RY2 | HSD17B1 | CYP24A1 | CCNE1 | HRH1 | CLK4 | PDCD4 |
| LIG1 | EGLN1 | GRIK2 | LGALS9 | P2RY6 | ADRA1A | SLC18A3 | TUBB1 | ADRA1B | PRKCZ | KCNA3 |
| PTGFR | EPHB4 | FDPS | FABP1 | CD22 | ITGA4 | KDM4A | PTK2B | GRIA1 | DRD4 | ITGB7 |
| SSTR5 | CHRNB2 | SLC1A2 | SLC1A1 | POLG | PIN1 | HSD17B3 | DBF4 | GRIA2 | SIGMAR1 | TKT |
| SSTR2 | PLG | GABBR2 | MMP8 | HDAC8 | BAZ2B | CTBP2 | CHRNB4 | KDM3A | CA3 | ADAM17 |
| SSTR4 | SOAT1 | GRM1 | CPA1 | RORC | BAZ2A | MAOB | FNTA | PGGT1B | HPGD | GRK1 |
| SSTR1 | SOAT2 | H1F0 | TRPM8 | CTSG | MTNR1A | LSS | ROCK2 | HTR5A | JAK1 | GLUL |
| SSTR3 | LANCL2 | CASR | MAP3K8 | CTRB1 | FASN | DHCR7 | OGFRL1 | LIMK1 | CSF1R | DPP4 |
| PPM1A | CDC45 | CTSC | HSPA1A | TDO2 | RARA | PLA2G2A | PNMT | CCR9 | CYP11B1 | SLC28A2 |
| TREH | NMUR2 | KCNK2 | IGF2R | DBH | FABP4 | CYP2C9 | SCN5A | PARP1 | CYP11B2 | TMIGD3 |
| HEXA | PLA2G2D | SLC6A12 | GPR17 | NFKB1 | FABP3 | CYP3A4 | SLC6A9 | P2RX7 | JAK3 | CCND1 |
| HEXB | PTK2 | PEPD | KDM5C | SNCA | KCNA5 | IGFBP5 | ADRA1D | PARP2 | CFD | MGAM |
| GLRA1 | ESRRG | TACR2 | KDM4B | DNM1 | CACNA1G | DNMT1 | ROCK2 | DNMT3A | HRH3 | IL2 |
| GLRA2 | SMPD2 | IL6 | RNPEP | GPBAR1 | HTR1D | ERN1 | PLA2G10 | CDK1 | MAPK14 | QARS |
| PYGB | PHF8 | TAAR1 | POLD1 | CPB2 | ATIC | KDM6B | VDR | HMGCR | PDE4B | CASP6 |
| AMY2A | GRM2 | PREP | TDP1 | CPN1 | CHRNA5 | FTO | KCNH2 | ADORA2B | RPS6KB1 | SLC5A4 |
| PPARA | CACNA2D1 | TXN | HSD17B7 | BRAF | MAPK9 | IGF1R | CYP2D6 | MTOR | ICAM1 | FPGS |
| GGH | GRM3 | TXN2 | PPP5C | FEN1 | PTPRC | TEK | ITGA2B | CA1 | SELE | MME |
| FABP5 | GRM6 | MCL1 | FGR | GPR84 | PLA2G4A | DPEP1 | ADRA2A | HPGDS | NOX4 | MAPK1 |
| PPARD | PGD | PRKCI | LYN | CBR1 | NQO2 | F3 | TRPA1 | AKR1C1 | WNT3A | HPRT1 |
| ERCC5 | GRM8 | EPHX2 | HK1 | NAT1 | TLR4 | HRH2 | UGT2B7 | ATM | MTNR1B | P2RY1 |
| DDO | GRM4 | SLC37A4 | PPP1CA | PLA2G4B | STS | NFE2L2 | CHRNA3 | PDE8B | EP300 | OPRM1 |
| CAD | HTR1B | MAG | FLT4 | COMT | CCR3 | STAT3 | ACE2 | CA4 | KAT2B | SETD7 |
| HAO1 | GSTK1 | LGALS4 | PLK1 | PTPRB | HDAC4 | PIM2 | FAAH | BRD4 | TNKS2 | NEU3 |
| TK1 | BCL2 | HK2 | MB | ALDH5A1 | MAPK11 | PRMT8 | SRD5A1 | AKR1C3 | CA5A | NEU2 |
| CDK9 | RARG | PTGES | RNASEH1 | ABAT | XIAP | PRMT6 | CHRM5 | FLT3 | SCN9A | UPP1 |
| QDPR | RARB | TNKS | GSR | FUT7 | NGFR | HTR7 | F10 | JAK2 | HIPK4 | OGA |
| PLK4 | ALK | KDR | POLK | HTR1F | FAP | RELA | SLC22A12 | CASP3 | SBK1 | FUCA1 |
| PIP4K2C | MAPK10 | GBA | CA5B | IARS | PIM1 | PRKCB | GLA | SLC6A1 | LDHA | KDM1A |
| ABL1 | MYLK2 | CHRM1 | CHRM3 | GANAB | SELL | STK3 | HSPA8 | RASGRP1 | LDHB | POLA1 |
| KIT | CSNK1D | GBA2 | PTGS1 | ERBB2 | SELP | CCNB2 | CDC25A | CPT1A | IL1B | CFTR |
| RET | ERBB4 | CA7 | PRKCA | FYN | PRKCG | HCAR2 | HSPA5 | CCR1 | FLT1 | CDA |
| BLK | IKBKB | GAA | ATP2A1 | OPRD1 | HTR4 | OPRL1 | HRH4 | SLC47A2 | PPM1B | MMP12 |
| HTR2C | RPS6KA4 | AGL | ADK | POLB | HSD11B1 | DRD1 | TTL | NMT1 | PPP1CC | GRIN1 |
| PHKG2 | HCK | SLC29A1 | DNMT3B | ADRB1 | NOS1 | RASGRP3 | TRPV4 | CHUK | PTAFR | CTSS |
| DAPK3 | IRAK1 | SI | PDE1B | GSTM1 | PTPN6 | PLCG1 | VAV1 | CPT1B | PPP2R5A | CTSE |
| ADORA2A | IRAK4 | MTAP | LGALS8 | MAP3K7 | NOS3 | FUCA2 | PCSK7 | TARS | KDM5B | TTR |
| CHEK2 | PRKD1 | CA6 | MAP2K1 | YARS | ADRB3 | GANC | MAN2B1 | MMP10 | DRD5 | CYP1B1 |
| VCP | PDE2A | ITGAL | CA9 | DAPK1 | AKR1C4 | MMP2 | MKNK2 | LIPG | NOTUM | TNF |
| SLC16A3 | PDE11A | ST3GAL3 | TP53 | HSD17B2 | CA13 | CYP26A1 | MKNK1 | F11 | CASP8 | TOP2B |
| NOD2 | PDE1A | MMP1 | CHEK1 | MMP3 | AKR1A1 | MAN1B1 | DOT1L | F7 | FOLH1 | SIRT2 |
| TERT | ACHE | ADA | EGFR | TYR | AMY1A | NTRK1 | MMP13 | ITGB1 | SPTLC1 | CHRNB1 |
| FN1 | PDE1C | ACLY | ESRRA | SLC5A1 | GRK6 | AKT1 | MMP7 | CHRNA3 | SPTLC2 | DRD2 |
| RXRA | SLC6A4 | DAO | MPG | SLC5A2 | HDAC3 | CES1 | F9 | ADAMTS4 | PRKCD | HDAC2 |
| AKR1B1 | DYRK1A | PYGM | SAE1 | PDE5A | HDAC6 | PRKDC | MAP3K14 | P2RY10 | PRKCE | BACE1 |
| FGF2 | HTR6 | ALPL | HSP90AA1 | SF3B3 | MAPT | NQO1 | SLC18A2 | FKBP1A | SCD | NCSTN |
| NADK | CLK1 | HPSE | CYP1A1 | PSMB5 | KDM4E | MAN2A1 | CRHR1 | ADRB2 | PRKCQ | CLK3 |
| HRAS | DYRK1B | PTPN2 | CYP1A2 | CXCR1 | KISS1R | HSP90AB1 | PTGES2 | OPRK1 | F2 | STK17A |
| PSENEN | HSP90B1 | CASP2 | SLC28A3 | CAMK2B | DNM2 | CYP2C19 | CA2 | TMPRSS15 | TLR9 | APH1A |
| XPO1 | NOS2 | LAP3 | EIF4A1 | NAMPT | MYLK | MLX | GCGR | FKBP4 | ITGAV | ATP1A2 |
| MGMT | EPHX1 | NEK6 | GPR35 | BRPF1 | KLK1 | P2RX3 | ADORA1 | MANBA | ITGB5 | CHRNA6 |
| PDGFRA | KDM2A | ANPEP | AVPR2 | GRIN2D | KLK2 | FABP2 | ALOX5AP | PTPN22 | CTSD | KCNJ10 |
| GAPDH | CTSK | CA14 | ASNS | CROT | APEX1 | PSEN2 | IMPDH1 | TNNC1 | HLA-A | GP9 |
| AKR1B10 | REN | GRIK1 | MAOA | SLC22A5 | CDK5R1 | AGTR1 | INSR | CCKBR | MLNR | HTR1A |
| ODC1 | CTSL | PON1 | TAS2R31 | ALB | CCNB3 | LCK | TRPV3 | DYRK2 | GLS | ADRA2C |
| TRPM2 | HDAC10 | TYMP | ABCC1 | CRAT | ARG1 | NR1H4 | PSEN1 | KCNJ14 | SIRT1 | ATP1A1 |
| HIF1A | DUSP3 | AMPD2 | PIM3 | CHRM4 | CDK6 | APH1B | PNP | TBXAS1 | NPY1R | KCNN4 |
| GRM5 | RAF1 | PIK3CD | ACVRL1 | ABCB1 | HDAC1 | FDFT1 | PRSS1 | ITGAV ITGB1 | DHFR | DRD3 |
| ABCG2 | TUBB3 | PIK3CB | PTP4A3 | SLC6A2 | PPP2CA | DPP7 | IGFBP3 | FOS JUN | C3AR1 | GABRB3 |
| NRP1 | NAALAD2 | PIK3CG | PPARG | GRIN1 | KDM4D | AGTR2 | LTA4H | ITGA2B | GRB2 | KCNK6 |
| MMP9 | MAP3K5 | ALDH2 | GGPS1 | SLCO1B1 | KDM4C | DPP9 | GHSR | ITGAV | ITGB1 | CHRND |
| FFAR1 | ENPEP | TBXA2R | KMT2A | PTPRA | FANCF | TYRO3 | LGALS1 | DDOST | MGLL | IMPDH2 |
| RARS | PTP4A2 | MIF | ADCY1 | GRIK3 | EPHA2 | MAP4K4 | MARS | EPAS1 | USP10 | BCHE |
| LGALS3 | PTP4A1 | CASP1 | SUV39H1 | ALOX15 | CHRNB4 | PTGDR2 | METAP2 | AURKA | USP13 | KCNJ1 |
| ITGA3 | EIF4H | RRM1 | INMT | PFKFB3 | MAP3K9 | BIRC2 | PRSS3 | LRRK2 | SERPINE1 | CHRNA9 |
| CAPN1 | TPMT | PTPN1 | SMYD2 | FUT4 | FGFR1 | FFAR4 | PKN1 | ADORA3 | PIK3CA | CHRFAM7A |
| GART | AHR | ADAMTS5 | EZH2 | STAT1 | HASPIN | BDKRB1 | CSNK2A1 | MCHR1 | TMPRSS11D | ITGAL |
| CTSB | KMO | ACE | EZH1 | PGF | BTK | JUN | SYK | ATR | P2RY12 | TUBAL3 |
| WEE1 | NEU4 | PRKAB1 | SETDB1 | CTSA | MMP16 | LNPEP | LTB4R | HTT | UGCG | CHRNA5 |
| PDE10A | P4HTM | PDYN | TPSAB1 | PDE4D | AHCY | MMP14 | NEK2 | HMOX1 | ST14 | SLC22A6 |
| CDC25B | CTNNB1 | PDE3A | CARM1 | XPNPEP1 | AMD1 | ERAP2 | PRKACA | LIPE | MDH1 | RORA |
| SHH | DTYMK | ELANE | PRMT1 | NTRK2 | HTR3A | ERAP1 | GPR34 | NUDT1 | S1PR3 | PTGDR |
| GSK3B | SLC13A5 | NTSR2 | EHMT1 | SLC10A2 | NR1I3 | CCNE2 | SPHK2 | QPCT | S1PR1 | ENPP2 |
| ESR1 | IKBKG | NTSR1 | EHMT2 | AXL | TOP2A | MAP2 | ITGA2 | PRKAG1 | MDH2 | ALOX12 |
| PDE9A | AMPD3 | F3 | GLO1 | NUAK1 | NR3C2 | CDC25C | SPHK1 | PRKAA2 | DGAT1 | PDE4A |
| CHRM2 | ECE1 | F7 | GPR55 | AKR1C2 | CYP26B1 | TOP1 | DPP8 | ALOX5 | PTGER2 | PTGER3 |
| PABPC1 | GNPAT | CA12 | MPO | XPNPEP2 | GLB1 | EDNRA | MET | SRC | ADRA2B | HNF4A |
| HTR2B | ENGASE | TYK2 | PIK3R1 | BCL2L2 | SLC2A1 | CDK1 | LIPC | BDKRB2 | GLI1 | SQLE |
| GCG | CPT2 | CYP19A1 | PTPRF | SRD5A2 | MST1R | ZAP70 | PTPA | PDPK1 | GABRG2 | SLC16A1 |
| SLC25A20 | KCNJ11 | NR1H3 | ACP1 | PGR | RGS4 | CCND3 | TYMS | MAPK8 | GABRA5 | CCNB1 |
| CHRNB3 | CHRNA1 | CNR1 | PTPN11 | HSD11B2 | HCRTR2 | SLC9A1 | PRMT3 | BRD2 | CDK2 | GABRG2 |
| GLRA3 | KCNJ15 | NR3C1 | PLA2G1B | AR | KCNE1 | KIF20A | SLC8B1 | BRDT | CCNE1 | GABRA1 |
| CACNA1I | SLCO1B3 | CD81 | ESR2 | SLC6A3 | PGK1 | HSF1 | CDK7 | CCNH | CHRNA6 | CDK2 |
| GRIN2A | GRIN2C | PRKCH | PTGIR | MAPK3 | CAPN2 | GABRB3 | GRK3 | CCND1 | TNNT2 | CDC7 |
| CD38 | PDGFRB | SERPINA6 | TRPV1 | PTGER1 | NPY5R | GCK | TSPO | FNTB | TNNI3 | CHRNA2 |
| PDE7A | CCNA1 | SHBG | GABRB2 | PTGER4 | NPBWR1 | GRK5 | FADS1 | CCND2 | ROCK1 | ITGB6 |
| SLC25A29 | CCNA2 | G6PD | NPC1L1 | CLK2 | PDE4C | CAMK2D | PKM | CHRNA4 | EBP | ITGB3 |
| CHRNA10 | CHRNE | CYP51A1 | ITGB2 | FPR2 | UBA2 | GRK2 | THRB | GRIN2B | GABBR1 | CDK5 |
| SLC22A4 | ICAM1 | PTGS2 | CYP17A1 | CMA1 | KCNQ1 | MECP2 | EPHB3 | ITGA5 | GABRB2 | CDK1 |
| CCNT1 |  |  |  |  |  |  |  |  |  |  |

Table S3: 173 targets related to rheumatoid arthritis.

| Targets | | | | | | |
| --- | --- | --- | --- | --- | --- | --- |
| ABCB1 | CASP3 | FGF2 | HSPA8 | MME | PGR | SLC1A2 |
| ACHE | CCR1 | FKBP1A | HTR2A | MMP1 | PIK3CG | SLC22A4 |
| ACLY | CCR9 | FLT1 | ICAM1 | MMP13 | PIN1 | SLC6A2 |
| ADA | CDA | FLT3 | IGFBP5 | MMP14 | PLA2G2A | SLC6A4 |
| ADAM17 | CDK6 | FN1 | IKBKB | MMP2 | POLA1 | SNCA |
| ADAMTS4 | CFD | FPGS | IL1B | MMP3 | POLB | SPHK1 |
| ADORA2A | CHEK2 | FPR2 | IL2 | MMP9 | POLD1 | SQLE |
| ADRA2A | CTSB | GABRA1 | IL6 | MPO | PON1 | SRC |
| ADRA2B | CTSC | GABRA5 | IMPDH1 | MTAP | PPARG | STAT1 |
| ADRA2C | CTSG | GABRB3 | ITGB2 | MTOR | PREP | STAT3 |
| ADRB2 | CXCR1 | GLUL | JAK1 | NAMPT | PRMT3 | SYK |
| AKR1A1 | CXCR2 | GRIN2A | JAK2 | NFKB1 | PTGER1 | TLR4 |
| ALB | CYP19A1 | GSR | JAK3 | NGFR | PTGER2 | TLR9 |
| ALOX12 | CYP1A2 | HCK | KCNJ15 | NR1H3 | PTGER3 | TNF |
| ALOX15 | DHFR | HDAC1 | KIT | NR3C1 | PTGER4 | TP53 |
| ALOX5 | DNM1 | HDAC2 | LAP3 | NRP1 | PTGES | TSPO |
| ALPL | DPP4 | HEXA | LDHB | NTRK1 | PTGS1 | TXN |
| ARG1 | DRD2 | HIF1A | LGALS1 | NTRK2 | PTGS2 | TYK2 |
| ATIC | EHMT2 | HLA-A | LRRK2 | OPRD1 | PTK2B | TYMS |
| ATM | ELANE | HMGCR | MAP4K4 | OPRK1 | PTPN1 | VDR |
| BLK | EPAS1 | HMOX1 | MAPK3 | OPRL1 | PTPN22 | VEGFA |
| BRD2 | EPHX2 | HPRT1 | MCL1 | OPRM1 | PTPRC | XIAP |
| C3AR1 | F10 | HRH2 | MDM2 | PARP1 | RORC |  |
| CA2 | FABP4 | HSP90B1 | MGLL | PGF | S1PR1 |  |
| CAMK2D | FDPS | HSPA1A/HSPA1B | MIF | PGK1 | SELE |  |
